# Neumann’s Hand Book of Skin Diseases

**Published:** 1872-01

**Authors:** 


*Neumann's Hand Book of Skin Diseases.* Translated from the German by Lucius D. Buckley, A.M., M.D. New York, D. Appleton & Co., 1872.

The frequent appearance of works upon skin diseases indicates the unsettled condition of this branch of medicine as well as the active efforts being made to remove the confusion which has always existed in the nomenclature and classification of skin diseases.

From a necessarily very imperfect examination of the work before us, we see that much careful investigation has been made to throw all possible light upon the microscopic changes which take place in many skin diseases, and to indicate as completely as may be, the best measures of relief. It is a work of practical value, and evidently is made to be concise, comprehensive and practical. The text is illustrated with finely made wood cuts, which adds greatly to a correct understanding of the pathology of many skin diseases. This book is for the careful student of skin diseases as well as text book for the active practitioner.

